# Ceiling culture of human mature white adipocytes with a browning agent: A novel approach to induce transdifferentiation into beige adipocytes

**DOI:** 10.3389/fbioe.2022.905194

**Published:** 2022-08-15

**Authors:** Yufei He, Zhuokai Liang, Jing Wang, Haojing Tang, Jian Li, Junrong Cai, Yunjun Liao

**Affiliations:** Department of Plastic and Cosmetic Surgery, Nanfang Hospital, Southern Medical University, Guangzhou, Guangdong, China

**Keywords:** white adipocytes, beige adipocytes, ceiling culture, transdifferentiate, metabolic disease

## Abstract

Excess and dysfunctional adipose tissue plays an important role in metabolic diseases, including obesity, atherosclerosis and type 2 diabetes mellitus. In mammals, adipose tissue is categorized into two types: white and brown. Adult brown tissue is mainly composed of beige adipocytes, which dispose of stored energy as heat and have become increasingly popular as a therapeutic target for obesity. However, there is still a paucity of cell models that allow transdifferentiation of mature white adipocytes into beige adipocytes, as seen *in vivo*. Here, we describe a novel, ceiling culture-based model of human mature white adipocytes, which transdifferentiate into beige adipocytes under the mechanical force and hypoxia of ceiling culture. We also show that the use of rosiglitazone and rapamycin can modulate transdifferentiation, up and down regulating expression of beige adipocyte-specific genes, respectively. Rosiglitazone additionally facilitated the upregulation of fatty acid lipolysis and oxidation genes. Finally, these beige adipocytes derived from dedifferentiated adipocytes exhibited a progenitor-specific phenotype, with higher expression of mature adipocyte-specific genes than adipocyte-derived stem cells. Overall, we report a novel approach to conveniently cultivate beige adipocytes from white adipocytes *in vitro*, suitable for mechanistic studies of adipose biology and development of cell and drug therapies in the future.

## Introduction

Obesity is an increasingly prevalent lifestyle disease defined by excess adipose tissue (Saxton et al., 2019). Adipose tissue is a highly plastic organ essential for energy homeostasis by storing and releasing fat (primarily triglyceride) in response to energy demands (Klöting and Bluher, 2014). The limited storage capacity of adipose tissue can lead to ectopic fat storage, a key element in the pathogenesis of insulin-resistant cardiometabolic diseases, which can result metabolic dysfunction and in an imbalance of lipid homeostasis (Lotta et al., 2017).

Inducing the browning of white adipose tissue (to create beige adipocytes) may become a way of treating obesity and its associated metabolic complications (Cheng et al., 2021). Beige adipocytes exhibit multilocular lipid droplets (LDs), similar to brown adipocytes *in vivo*, and possess the metabolic activity of thermogenic cells, such as uncoupled respiration and cAMP-induced lipolysis (Cannon and Nedergaard, 2004). Compared with white adipocytes, beige adipocytes promote glucose clearance and fatty acid oxidation, thereby increasing energy consumption *via* thermogenesis, and potentially protecting against obesity and metabolic dysfunction (Desjardins and steinberg, 2018; Yao et al., 2017). As a result, an attractive therapeutic strategy has been identified to transform mature unilocular white adipocytes into multilocular, brown-like adipocytes with high uncoupling protein 1 (UCP1) expression (Harms and Seale, 2013).

However, the origin of beige adipocytes is controversial: they may transdifferentiate from existing mature white adipocytes or differentiate directly from beige precursor cells (Kaisanlahti and Glumoff, 2019; Wang et al., 2013; Wu et al., 2012). The browning of white adipose tissue by drug-induced transdifferentiation has become an increasingly popular research focus, including *via* peroxisome proliferator-activated receptor (PPAR)γ agonists (Rosiglitazone), β3 adrenergic receptor agonists (Mirabegron), and cyclic guanosine monophosphate-dependent protein kinase I agonists (Sildenafil) (Cypess et al., 2015; Merlin et al., 2018; Mitschke et al., 2013). However, due to a lack of appropriate culture models of transdifferentiation *in vitro*, most cell models use beige adipose precursor cells induced to differentiate into beige adipocytes (Hammarstedt et al., 2018; Rui, 2017). Therefore, a cell model of transdifferentiation of mature white adipocytes into beige cells is required.

Models of mature white adipocytes *in vitro* are still poorly understood, potentially slowing the development of clinical treatments for type 2 diabetes mellitus and other metabolic diseases. A method that utilizes a permeable, porous membrane and allows culture of freshly isolated, mature adipocytes provides a more representative model of *in vitro* conditions (Harms et al., 2019); however, the effect of oxygen concentration is often not considered on the fragile white adipocytes separated from the body. Another widely used method uses the stromal vascular fraction (SVF) from isolated adipose tissue and a 3D biological scaffold to create an adipose depot *in vitro*, termed “fat-on-a-chip” (Bender et al., 2020). Although this model maintains the structural integrity and functional characteristics of adipose tissue, such as lipolysis and secretion, it still differs from mature adipocytes *in vivo*.

Due to these limitations, many researchers rely on adipocytes differentiated from various cell types, including mouse embryonic fibroblasts, SVF from adipose tissue and the 3T3-L1 fibroblast-like cell line. However, it is unclear how much these cells represent the adipogenesis process from precursor cells *in vivo* (Ghaben and Scherer, 2019). As a result of the shortcomings of current cell models, development of cell and drug therapies for obesity and its related diseases has been severely restricted.

One method to overcome the limitations of current models is the ceiling culture method. During the process of transdifferentiation of white adipocytes to beige adipocytes, an increase of mitochondria and formation of numerous small LDs occurs (Barbatelli et al., 2010; Himms-Hagen et al., 2000). During this process, physical mechanics can facilitate the reduction of LD volume *via* lipolysis and liposecretion, allowing fatty acids to consumed as an energy source. The ceiling culture method applies mechanical force on freshly isolated, mature adipocytes, which float on culture medium and attach to the top of a filled, inverted cell culture flask (the “ceiling”). A few days later, adhered cells rapidly transdifferentiate into fibroblast-like cells, which are named dedifferentiated adipocytes (DAs) (Sugihara et al., 1987, 1986). Previously, we found a higher retention rate of fat grafts by using beige adipocytes from ceiling culture than adipose-derived stem cells (ADSCs), suggesting cells cultured by this method are physiologically relevant model (Xia et al., 2021).

In present, there are a number of characteristic specific markers are investigated during the period of adipocyte differentiation and maturation. Peroxisome proliferator-activated receptorγ (*PPARγ*), a primary regulator in adipocyte differentiation and maintenance, governs the main gene expression of lipid and glucose metabolism such as adipogenesis and fatty acid transport (Hernandez-Quiles et al., 2021). Its expression was detected in most of the induction processes in present study, which revealed cell component changes after applied agents. Expression of glucose transporter type 4 (*GLUT4*) has been demonstrated increased insulin-stimulated glucose uptake after the adipocyte differentiation. In addition, as a significant role in energy and expenditure, the adipocyte hormone Leptin is referred in control of adipocyte differentiation and homeostasis (Hwang et al., 1997). Adipocyte lipid binding protein 2 (*AP2*, also known as *FABP4*) is recognized as an essential link between lipid metabolism and cellular functions in adipocytes, which promotes proteosomal degradation of *PPARγ* in the terminal stage of adipocyte differentiation (Yang and Smith, 2007). These lineage specific differentiation markers were better to assess triglyceride accumulation per cell and adipocyte-specific gene expression during the process of transdifferentiation.

In this report, we describe a novel method for the ceiling culture of mature white adipocytes combined with a browning inducer (Rosiglitazone), which causes transdifferentiation into beige adipocytes. These adipocytes provide an *in vitro* model that simulates the phenotype and function of beige cells *in vivo*, which was superior to traditional culture methods. Importantly, we found evidence that mature white adipocytes can directly transdifferentiate into beige cells *in vitro* without the involvement of precursor cells.

## Materials and methods

### Human adipose

Adipose tissue was removed as abdominal lipoaspirates from three young, non obese females (mean BMI = 22.8 ± 0.26). Under general anesthesia, the abdominal wall was injected with saline solution containing 0.001% epinephrine and adipose tissue was suctioned using a multiport 3 mm cannula with sharp side holes 1 mm in diameter. Freshly obtained fat tissue was preserved at 4°C and prepared for downstream experiments.

### Isolation and induction of beige adipocytes and ADSCs

Approximately 20 g of adipose tissue was incubated for 45 min at 37°C with 0.075% (w/v) collagenase solution (collagenase type I; Sigma, St. Louis, MO, United States); tissue was rotated two to three times at 15 min intervals. After 45 min, collagenase digestion was terminated by the addition of an equal volume of complete medium consisting of high-glucose (4.5 g/L) Dulbecco’s Modified Eagle Medium (Gibco, Thermo Fisher Scientific, Inc., Waltham, MA, , United States), 10% (v/v) fetal bovine serum (Gibco), and 1% penicillin-streptomycin solution (Gibco); the cell suspensions were then centrifuged for 10 min at 200 rpm. Floating, unilocular adipocytes were harvested from the top layer and filtered with 100 µm strainer; ADSCs were harvested from the SVF in the bottom layer and filtered with 100 and 70 µm strainers (Xia et al., 2021). Harvested ADSCs was identified the surface antigens CD44 by flow cytometry and detected multi-differentiation potential (adipogenic, osteogenic, and chondrogenic characteristics). Adipocytes and ADSCs were respectively identified by qPCR to detect the expression of *FABP4*, *SLC2A4* and *CD44*, *THY1* markers (Wheeler et al., 2018) (Supplementary Material S1).

For ceiling culture of beige adipocytes, extracted mature adipocytes were seeded at a density of 0.5–1 × 106 cells per 25 cm^2^ flask. Flasks were filled with complete culture medium and inverted, so that floating cells could attach to the top surface of the flask. Culture medium consisted of high-glucose (4.5 g/L) Dulbecco’s Modified Eagle Medium, 10% (v/v) newborn calf serum, 4 mM L-glutamine, 50 U/ml penicillin, and 50 U/ml streptomycin; cells grown in this condition are referred to as ceiling-cultured cells. Where indicated, this was supplemented with 1 µM rosiglitazone (Merlin et al., 2018); cells grown in this condition are referred to as ceiling-cultured+roziglitazone cells. Cells were incubated at 37°C in 5% carbon dioxide; after 7 days, flasks were reinverted, and medium was changed every 3 days.

To culture and induce the ADSCs, cells from the SVF were seeded into a 25 cm^2^ flask and cultured in Growth Medium for Human ADSCs (Cyagen Biosciences, Inc., Guangzhou, People’s Republic of China) supplemented with 10% (v/v) fetal bovine serum, 2 mM L-glutamine, 50 U/ml penicillin, and 50 U/ml streptomycin and subcultured after reaching 70–80% confluence. After cells were passaged one to three times, confluent cultures were stimulated with a “browning cocktail” (growth media supplemented with 0.5 μg/ml insulin, 10 µM dexamethasone, 0.5 mM 3-Isobutyl-1-methylxanthine, 1 µM rosiglitazone, and 2 nM triiodothyronine) for 3 days. After this, cells were maintained in growth media supplemented with 0.5 μg/ml insulin for 1 day. The process of stimulated and maintained culture was continuously repeated three to four times (12–16 days). Cells were treated with CL316,243 (2 μM, Apexbio, Houston, United Kingdom), a highly selective β3-adrenoceptor agonist, 8–12 h before harvest.

### Transmission electron microscopy

Beige adipocytes differentiated from ADSCs, ceiling culture-induced beige adipocytes, DA-induced beige adipocytes and DA-intermediate state (multilocular adipocytes formed in ceiling-cultured with a similar appearance to beige adipocytes) were washed with phosphate-buffered saline (PBS) and centrifuged at 3000 rpm for 5 min to form a pellet. The cell pellet was fixed with 2% glutaraldehyde in PBS for 2 h at 4°C, then postfixed by incubating with 1% osmium tetroxide in phosphate buffer 0.1 M (pH 7.4) for 15 min three times. Finally, the sample was dehydrated in ethanol and embedded in an Epon-Araldite mixture. Thin sections were obtained with an EM UC7 Ultramicrotome (Leica, Wentzler, Germany), before staining with lead citrate, and examining with a transmission electron microscope (Tecnai G2 Spirit 120kV; ThermoFisher, NY, United States). For each condition, 100 cells were randomly picked and examined at a final magnification of 8750×. The cytoplasmic area was obtained with the aid of an image analysis system (Tecnai G2 Spirit 120kV; ThermoFisher, NY, United States). The investigators performing the transmission electron microscopy were blinded to the study and were asked to make an unbiased assessment.

### MTT assay

Cell viability was determined by using the 3-(4,5-dimethylthiazol-2-yl)-2,5-diphenyltetrazolium bromide (MTT; Solarbio, Guangzhou, People’s Republic of China) colorimetric assay. In short, cells were seeded into 96-well plates and treated for 72 h with different concentrations of rapamycin (0, 10, 25 and 100 nM). After treatment, cells were washed with PBS and incubated in MTT solution (Solarbio) for 4 h. Dimethyl sulfoxide was then added to each well, and absorbance was measured at 490 nm with a microplate reader (BioRad, CA, United States).

### Oil Red O staining

Cells were fixed with 10% formalin and incubated for 30 min at 37°C with gentle shaking. Cells were then washed with 60% isopropanol and incubated in Oil Red O working solution. After 10 min, plates were rinsed with distilled water four times. Next, cells were stained in Mayer’s hematoxylin for 30 s and washed thoroughly in distilled water three times. Oil Red O was quantified by measuring optical absorbance at 510 nm.

### Alizarin Red staining

Cells were fixed in cold 10% formalin for 20 min at 4°C. Fixed cells were then stained with 400 µl Alizarin Red at pH 7.2 for 5–10 min. For calcium quantification, 300 µl 10% acetic acid was added to cells stained with Alizarin Red and incubated for 30 min at room temperature with agitation. Cells were scraped off and centrifuged for 30 min at 2000 rpm, before 200 µl of the supernatant was transferred to another microtube, and 22.5 µl of 10% ammonium hydroxide was added to neutralize the acid. Finally, optical absorbance was measured at 405 nm.

### Alcian Blue staining

Cells were fixated in 10% formalin for 1 h and washed twice with PBS before incubation in 1% Alcian Blue prepared in 0.1 N HCl for 30 min. Cells were washed three times in 0.1 N HCl and distilled water was used to neutralize the pH. Quantification was performed by incubation in 6 M Guanidine-HCl solution overnight at 4°C. Staining was measured *via* absorbance at 600 nm.

### Immunofluorescent staining

For immunofluorescent staining, cells were fixed with 4% paraformaldehyde and blocked with 5% BSA for 2 h at room temperature, and then incubated with the primary antibody rabbit anti-mouse UCP1 (dilution, 1:200; Proteintech, Chicago, United States). After washing, sections were incubated with the secondary antibody Alexa Fluor 488-conjugated goat anti-rabbit immunoglobulin G (1:200; Thermo Fisher, Holtsville, NY, United States). Nuclei were stained with DAPI (Solarbio, Guangzhou, People’s Republic of China).

### Western blot analysis

Whole beige adipocyte lysates (25 μg) in RIPA buffer containing protease inhibitor cocktail and phenylmethylsulfonyl fluoride were prepared using 5× SDS-PAGE loading buffer [250 mM Tris-HCl (pH 6.8), 0.25% bromophenol blue, 50% glycerol, 10% SDS, 0.5 M DTT (Bioseang Inc., Seongnam, Korea)] for separation by SDS-PAGE. After electrophoretic separation, gels were transferred onto activated polyvinylidene difluoride membranes for 1 h at 0.4 A. Membranes were blocked with 5% BSA for 2 h at room temperature and incubated with the primary antibody, rabbit anti-mouse UCP1 (dilution, 1:500; Proteintech, Chicago, United States)., which was diluted in blocking solution, for a minimum of 12 h at 4°C. Membranes were then incubated with secondary horseradish peroxidase-conjugated antibodies, HRP-conjugated affinipure goat anti-mouse immunoglobulin G (1:10,000; ZSGB-BIO, Inc., Beijing, People’s Republic of China)) for 2 h at room temperature, before visualization using the Supernova ECL western blotting detection system and imaging with the ChemiDocTMTouch (BioRad, CA, United States). Band intensities were quantified using ImageJ software. Assays were performed with samples from at least three independent experiments.

### Quantitative real-time polymerase chain reaction

Total RNA was extracted from cells using the RNeasy Lipid Tissue Mini Kit (Qiagen, Hilden, Germany), according to the manufacturer’s instructions. cDNA was synthesized and amplified over 40 cycles using a QuantiTect Reverse Transcription Kit (Qiagen) and a Rotor-Gene 3000 Real-Time PCR Detection System (Corbett Research, Sydney, Australia). (quantity of total RNA(ng) and cDNA(ng) were listed in Supplementary Material S2).

The primer sequences were as follows: UCP1: forward 5′-AGG​ATC​GGC​CTC​TAC​GAC​AC-3′ and reverse 5′-GCC​CAA​TGA​ATA​CTG​CCA​CTC-3′; PRDM16: forward 5′-CGA​GGC​CCC​TGT​CTA​CAT​TC-3′ and reverse 5′-GCT​CCC​ATC​CGA​AGT​CTG​TC-3′; PPARG: forward 5′-GGG​ATC​AGC​TCC​GTG​GAT​CT-3′ and reverse 5′-TGC​ACT​TTG​GTA​CTC​TTG​AAG​TT-3′; SOX9: forward: 5′-AGG​TGC​TCA​AAG​GCT​ACG​AC-3′ and reverse 5′-GTA​ATC​CGG​GTG​GTC​CTT​CT-3′; RUNX2: forward 5′-GGA​CGA​GGC​AAG​AGT​TTC​AC-3′ and reverse 5′-TGC​CTG​CCT​GGG​GTC​TGT​AA-3′; LIPE: forward 5′-CTC​CTC​CTA​TTC​CTA​ATC​CTC​C-3′ and reverse 5′-CAC​TTC​CTC​TTG​GGT​TTC​ACT​C-3′; PLIN1: forward 5′-GCA​AGA​AGA​GCT​GAG​CAG​AC-3′ and reverse 5′-AAT​CTG​CCC​ACG​AGA​AAG​GA-3′; CPT1A: forward 5′-AGA​ACA​CTC​ATG​GGC​AGA​TGC​T-3′ and reverse 5′-TAC​CTT​TCA​CCT​GGG​CTA​CAC​G-3′; ACACA: forward 5′-GGA​CCA​CTG​CAT​GGA​ATG​TTA​A-3′ and reverse 5′-TGA​GTG​ACT​GCC​GAA​ACA​TCT​C-3′ FABP4: forward 5′-GCT​TTT​GTA​GGT​ACC​TGG​AAA​CTT-3′ and reverse 5′-ACA​CTG​ATG​ATC​ATG​TTA​GGT​TTG​G-3′ SLC2A4: forward 5′-TGG​GCG​GCA​TGA​TTT​CCT​C-3′ and reverse 5′-GCC​AGG​ACA​TTG​TTG​ACC​AC-3′ CD44: forward 5′-CTG​CAG​GTA​TGG​GTT​CAT​AG-3′ and reverse 5′-ATA​TGT​GTC​ATA​CTG​GGA​GGT​G-3′ THY1: forward 5′-ATG​AAC​CTG​GCC​ATC​AGC​A-3′ and reverse 5′-GTG​TGC​TCA​GGC​ACC​CC-3′.

The expression of each gene was normalized relative to that of *GAPDH*, with expression levels calculated using the 2^−ΔΔCt^ method.

### Statistical analysis

Statistical analyses were performed using SPSS version 25.0 (IBM, Inc., Armonk, NY, United States). Data are expressed as mean ± SD and were compared among groups using a one-way analysis of variance or the Kruskal-Wallis test. Comparisons between two groups were performed using the least significant difference method or the Mann-Whitney *U*-test. A value of *p* < 0.05 was considered statistically significant.

## Results

### Browning occurs during dedifferentiation of white adipocytes in ceiling culture

**FIGURE 1:**
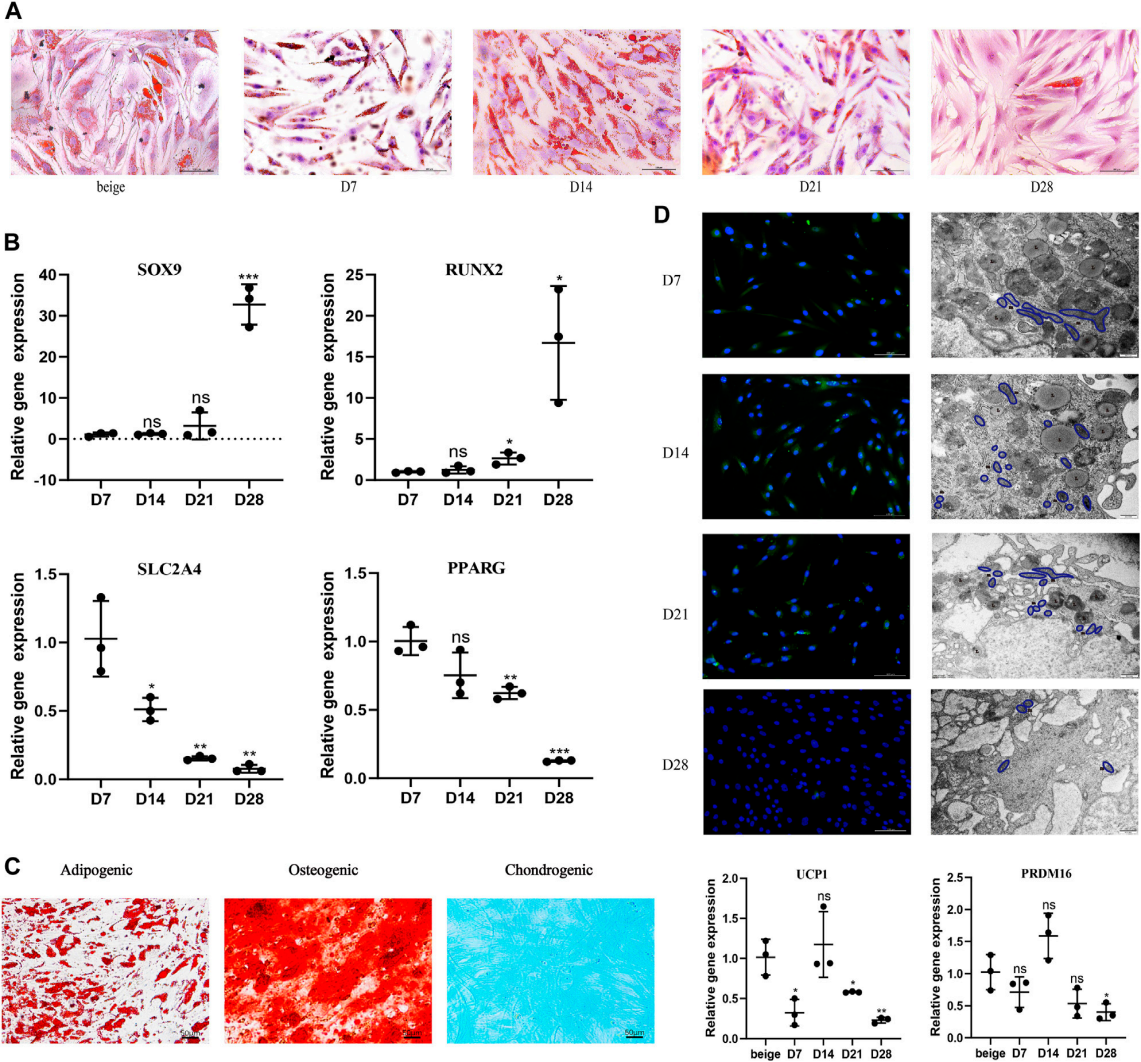
White adipocytes grown in ceiling culture display characteristics of beige adipocytes. **(A)** Intracellular lipid droplets were stained with Oil Red O and visualized by light microscopy at indicated timepoints (days) after the ceiling culture of mature white adipocytes. Scale bar 100 µm. **(B)** Relative gene expression levels of *SOX9*, *RUNX2*, *SLC2A4*, and *PPARG*, normalized to *GAPDH* as an endogenous control. **(C)** Human dedifferentiated adipocytes (DAs) were cultured for 3 weeks in adipogenic medium, osteogenic medium or chondrogenic medium. Cells were stained positively for lipid vacuoles with Oil Red O, for calcified extracellular matrix with Alizarin Red, and for chondrogenic differentiation Alcian Blue. Scale bar 50 µm. **(D)** Fluorescence microscopy visualized UCP1 expression stained with Alexa Fluor 488 (green) and nuclei stained with DAPI (blue). Scale bar 100 µm. Transmission electron microscopy showed condensed mitochondria and lipid droplet content at different stages of dedifferentiation. L, lipid; m, mitochondria. Scale bar 500 nm. Relative gene expression levels of *UCP1* and *PRDM16* compared to beige adipocytes, normalized to *GAPDH* as an endogenous control. Data are mean ± SEM (*n* = 3). ns, non-significant. *p < 0.05, **p < 0.01, ***p < 0.001.

After seeding, mature unilocular white adipocytes extracted from abdominal fat tissue of nonobese females successfully adhered to the flask in ceiling culture. Filling the flask with media also simulated the hypoxia and pressure seen *in vivo* after fat grafting. ADSCs from the same participant were induced and differentiated into beige adipocytes using the browning cocktail. To explore dedifferentiation capacity and timing *in vitro*, the appearance and quantity of LDs were observed 7, 14, 21, and 28 days post-seeding in ceiling culture with Oil Red O (Figure 1A). Compared with the positive control (ADSC-derived beige adipocytes), by D7, some white, dedifferentiating adipocytes showed formation of small, multilocular LDs., By D14, LD number increased and size decreased in all dedifferentiating adipocytes. At D21, the majority of cells had no visible LDs and finally differentiated into fibroblast-like cells, or DAs, at D28.

Previous research suggests DAs have high proliferative activity and a cell surface antigen profile similar to ADSCs; in addition, they retain or gain the expression of mesenchymal lineage-committed marker genes such as *RUNX2* and *SOX9* (Matsumoto et al., 2008). Dedifferentiation would also be expected to downregulate proadipogenic markers, such as PPARγ, which subsequently downregulates downstream expression of early adipogenic markers (Cawthorn et al., 2012). To examine potential changes in gene expression during different stages of ceiling culture, real-time PCR was used to analyze the expression of several markers in DAs and mature adipocytes. Expression of *RUNX2* and *SOX9* was higher, whereas expression of *SLC2A4*, which encodes the GLUT4 protein and is downstream of PPARγ (Benchamana et al., 2019), was lower, as was PPARγ mRNA (*PPARG*) at D28 than at D7 (Figure 1B).

DAs can also differentiate into adipocytes, chondrocytes, and osteoblasts under appropriate culture conditions *in vitro* (Matsumoto et al., 2008). We cultured DA cells with adipogenic, osteogenic, and chondrogenic induction medium and found, consistent with other researchers, abundant accumulation of LDs after 3 weeks of adipogenic induction. Osteoblast and chondrocyte characteristics were observed by staining for Alizarin Red or Alcian Blue, respectively; both of which were positive (Figure 1C). These results indicate that DAs obtained after ceiling culture are able to gain the characteristics of adipogenic-, osteogenic-, and chondrogenic-lineage cells and lose expression of mature adipocyte-specific markers.

We found that multilocular LDs formed in ceiling-cultured adipocytes had a similar appearance to beige adipocytes; we termed these DA-intermediate state cells. Beside LD size, we measured UCP1 protein expression by immunofluorescence during different states of dedifferentiation: UCP1 expression was higher at first, then decreased, with the peak appearing at D14. Transmission electron microscopy results showed that mitochondria content followed the same tendency, along with the decrease of LDs. We measured gene expression of transcription factors mainly expressed in mature brown/beige adipocytes, rather than SVF, which contains preadipocytes and other cell types (Seale et al., 2007). The expression of browning-related genes in ceiling-cultured adipocytes also peaked at D14, in line with the immunofluorescence results. However, the level of *UCP1* and *PRDM16* expression in DA-intermediate cells was partly lower than in beige adipocytes differentiated from ADSCs (Figure 1D). Although, ceiling-cultured cells displayed comparable *PPARG* expression at D7 and D14 (Figure 1B), LD morphology began to change, with large LDs at D7 reducing in size by D14 into smaller, multilocular droplets, most likely as a result of downregulation of early adipogenic factors.

### The dedifferentiation process is affected by browning of white adipocytes in ceiling culture

Previously, the mTOR pathway was identified as essential in the control of β3-adrenoceptor-stimulated glucose uptake in brown adipose depots (Olsen et al., 2014). However, rapamycin, the mammalian target of mTOR inhibitor, has been used as an alternative immuno-suppressive agent in clinical practice to minimize the side effect of some classical immuno-suppressive agents. The impact of rapamycin on insulin signaling, thermogenic gene expression, and mitochondrial respiration in brown/beige adipocytes has been well studied (García-Casarrubios et al., 2016). Thus, brown/beige adipocytes are established targets of rapamycin.

**FIGURE 2:**
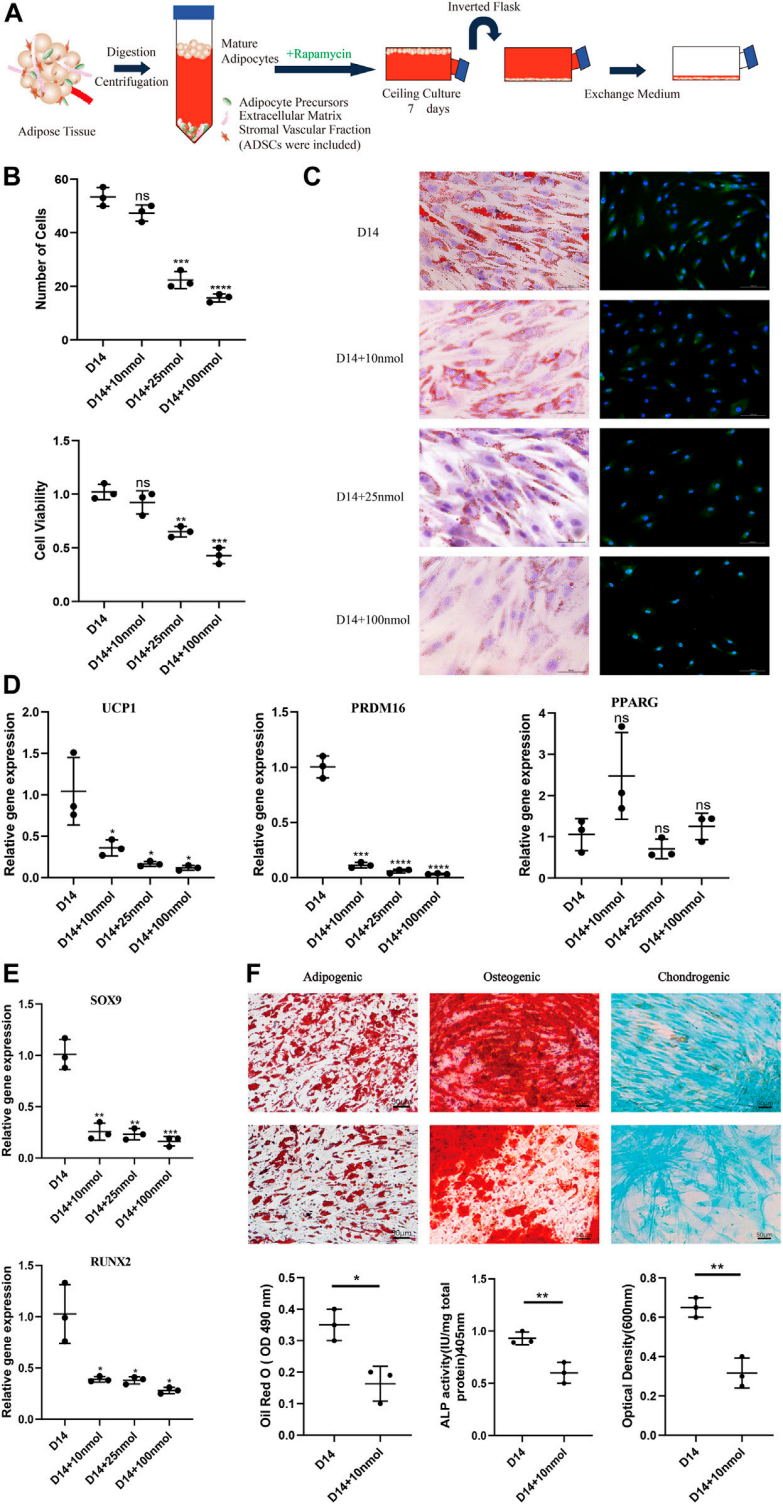
Effect of rapamycin on the dedifferentiation capacity of mature white adipocytes. **(A)** Model depicting the use of rapamycin to culture mature adipocytes. **(B)** MTT assay results confirmed that cell viability of dedifferentiated adipocyte (DA)-intermediate cells was inhibited by rapamycin, and the effect was concentration-dependent. **(C)** Intracellular lipid droplets were stained with Oil Red O and visualized by light microscopy. Fluorescence microscopy visualized UCP1 expression stained with Alexa Fluor 488 (green) and nuclei stained with DAPI (blue), in cells treated with 10, 25, and 100 nM rapamycin during ceiling culture of mature white adipocytes for 14 days. Scale bar 100 µm. **(D)** Relative gene expression levels of *UCP1*, *PRDM16*, and *PPARG*, normalized to *GAPDH* as an endogenous control. **(E)** Relative gene expression levels of *SOX9* and *RUNX2* normalized to *GAPDH* as an endogenous control, treated with 10, 25, and 100 nM rapamycin during ceiling culture of mature white adipocytes for 14 days. Data are mean ± SEM (*n* = 3). ns, non-significant. *p < 0.05, **p < 0.01, ***p < 0.001. **(F)** Confluent human DA cells were cultured for 3 weeks and treated with 10 nM rapamycin in adipogenic medium, osteogenic medium and chondrogenic medium. Cells were stained positively for lipid vacuoles with Oil Red O, before Oil Red O extracted from cells with isopropanol and quantified at an optical absorbance of 510 nm. Calcified extracellular matrix was visualized with Alizarin Red and measured at an optical absorbance of 405 nm. Chondrogenic differentiation was visualized with Alcian Blue and the extract was measured at 600 nm. Scale bar 50 µm.

To study the impact of the browning process on dedifferentiation modulation, we ceiling-cultured white adipocytes with or without rapamycin (at 10, 25, and 100 nM), with medium changed every 3 days (Figure 2A). When treated with 10 nM rapamycin, cells displayed minimal signs of toxicity and cell death; however, at higher doses, rapamycin treatment reduced cell number and adversely affected cell morphology (Figure 2B). At D14, cell viability was significantly and dose-dependently lower in the 25 and 100 nM rapamycin-treated groups than in cells treated with 10 nM rapamycin (*p* < 0.05). Thus, 10 nM rapamycin caused relatively low toxicity in these cells.

In addition, the number of LDs decreased in all rapamycin-treated cells and were barely visible in cells treated with 100 nM rapamycin. Immunofluorescence analysis showed that UCP1 expression decreased throughout the ceiling culture process in all rapamycin-treated groups compared with the untreated DA-intermediate adipocytes (Figure 2C). Compared with non-treated cells, rapamycin-treated cells displayed more than a three-fold decrease in expression of key browning genes, such as *UCP1* and *PRDM16* at D14, with no significant changes in expression of the adipogenic marker *PPARG* (Figure 2D). We also analyzed the expression of several cell lineage-specific markers in DA cells such as *RUNX2* and *SOX9*, which are critical transcription factors for osteogenesis and chondrogenesis, respectively. Expression levels of both these genes dose-dependently decreased as rapamycin concentration increased (Figure 2E).

Next, we investigated the effect of rapamycin on the function of DAs to further identify the relationship between browning and dedifferentiation. We chose DA cells cultured under different induction media and additionally added 10 nM rapamycin in the experimental group. Three weeks after adipogenic induction, accumulation of LDs in cells was lower in rapamycin-treated cells that untreated cells. In addition, mineralized matrix aggregates were observed with Alizarin Red staining, with this osteoblast marker attenuated in rapamycin-treated cells. Alcian Blue staining indicated a gradual decrease of sulfated proteoglycans and dispersion of the cells with rapamycin treatment (Figure 2F). These results suggest that rapamycin downregulates DA lineage commitment, with impaired functional characteristics of adipogenic, osteogenic, and chondrogenic cells.

### Ceiling-cultured human mature white adipocytes can transdifferentiate into beige adipocytes after browning induction

In recent years, attention has focused on the thermogenic action of brown/beige adipocytes in mammals in response to cold stimuli or β3-adrenergic agonists (Vitali et al., 2012). One study reported that beige adipocytes are highly inducible and display many similar characters compared to brown adipocytes, such as multilocular lipid droplets, dense mitochondria, and expression of UCP1.(Rui, 2017). There is evidence that under browning stimuli, some mature adipocytes can transdifferentiate into beige adipocytes (Jeremic et al., 2017; Jiang et al., 2017), although this is not conclusive.

**FIGURE 3:**
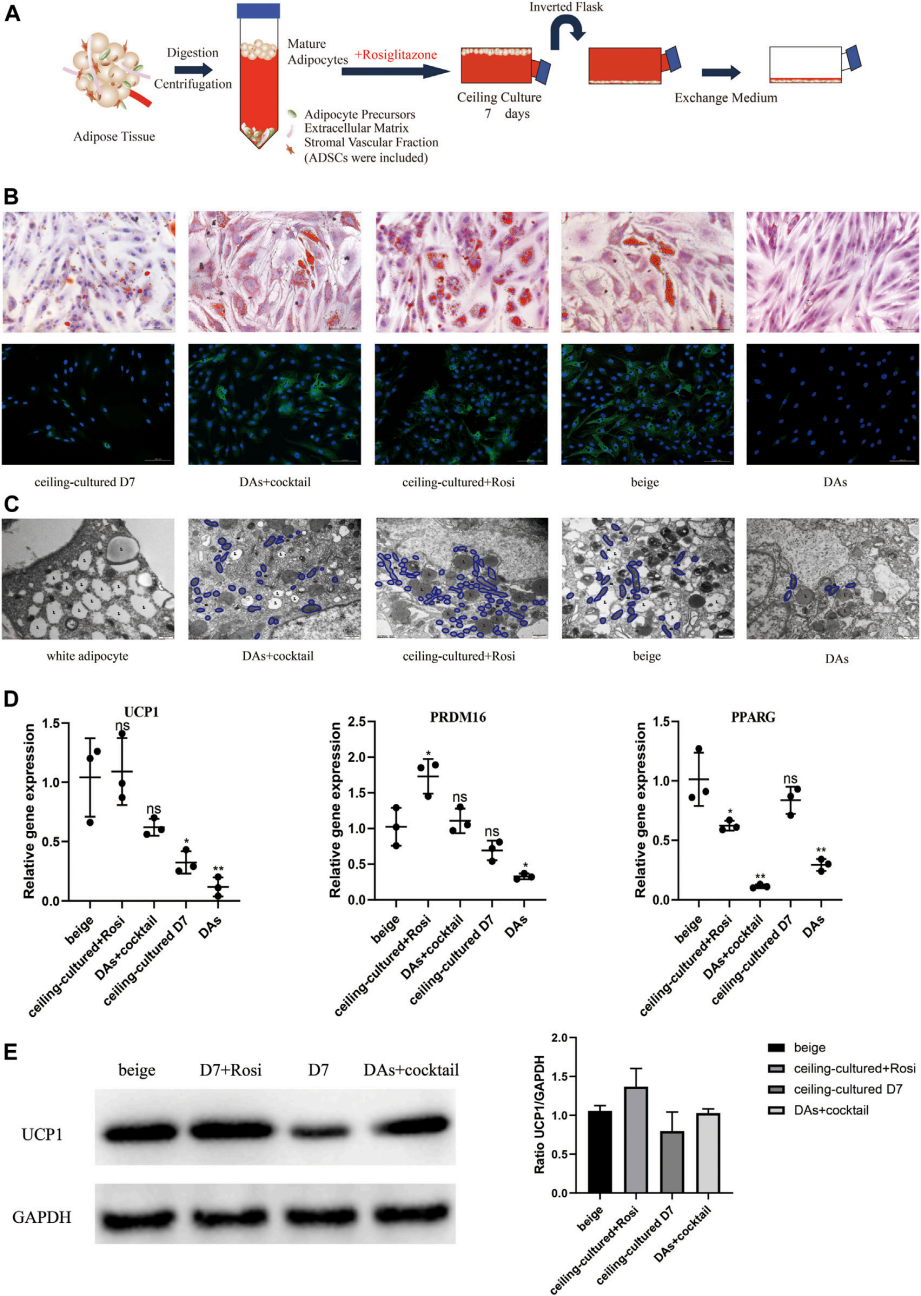
Ceiling-cultured adipocytes can transdifferentiate into beige adipocytes. **(A)** Model depicting the use of rosiglitazone to culture mature adipocytes. **(B)** Intracellular lipid droplets were stained with Oil Red O and visualized by light microscopy. Fluorescence microscopy visualized UCP1 expression stained with Alexa Fluor 488 (green) and nuclei stained with DAPI (blue). Scale bar 100 µm. **(C)** Transmission electron microscopy results showed that induced beige adipocytes and ceiling-cultured+Rosi cells exhibited condensed mitochondria compared with dedifferentiated adipocytes (DAs) and white adipocytes. L, lipid; m, mitochondria. Scale bar 500 nm. **(D)** Relative gene expression levels of *UCP1*, *PRDM16*, and *PPARG*, normalized to *GAPDH* as an endogenous control. Data are mean ± SEM (*n* = 3). ns, non-significant. *p < 0.05, **p < 0.01, ***p < 0.001. **(E)** Representative protein bands of UCP1. Corresponding peak areas were normalized to GAPDH, and relative ratios compared to GAPDH are shown. Data are mean ± SEM (*n* = 3). *p < 0.05, **p < 0.01, ***p < 0.001.

We developed a novel model whereby ceiling culture of mature white adipocytes directly transdifferentiate into beige adipocytes with induction by browning factors. We selected white adipocytes that had been in ceiling culture for 7 days and treated them with 1 µM rosiglitazone to explore their transdifferentiation capacity and efficiency compared with untreated ceiling-cultured cells (Figure 3A). In addition, ADSCs and DAs from the same donor were induced into multilocular “brown-like” adipocytes by the browning cocktail for comparison.

Characteristics of beige adipocyte transdifferentiation at D7 or D12 across the five cell types is shown in Figure 3B. Smaller, multilocular LDs were found in beige adipocytes (ADSCs+cocktail), DAs+cocktail and ceiling-cultured+rosiglitazone groups, whereas untreated ceiling-cultured D7 cells and DAs showed fewer or no visible droplets, most likely due to a lack of browning factors. We also measured UCP1 expression *via* immunofluorescence to assess the level of browning. UCP1 expression was significantly lower in DAs whereas expression was not different between DAs+cocktail beige cells and ADSCs+cocktail beige adipocytes at D12. Surprisingly, we found that ceiling-cultured+rosiglitazone cells expressed high levels of UCP1, comparable to classic beige adipocytes (ADSCs+cocktail), compared with untreated ceiling-cultured D7 cells.

Transmission electron microscope results showed that the three types of beige adipocytes (ADSCs+cocktail, DAs+cocktail and ceiling-cultured+rosiglitazone) contained more condensed mitochondria than white adipocytes, a trend replicated by UCP1 expression, where expression was higher in induced beige cells than untreated cells (Figure 3C). To validate the efficiency of various browning induction methods and assess the effect of rosiglitazone on browning markers, we measured gene expression of transcription factors involved in browning-related transdifferentiation. Interestingly, the expression of the browning-related genes *UCP1* and *PRDM16* in the ceiling-cultured+rosiglitazone group was the same or higher than beige adipocytes induced from ADSCs. Untreated ceiling-cultured D7 cells and beige adipocytes induced from DAs had lower expression of *UCP1* and *PRDM16* than the two groups above. Furthermore, ceiling-cultured D7 cells showed higher expression of *PPARG* than ADSC beige adipocytes, most likely due to the differences in cell origin (Figure 3D). Corresponding UCP1 protein levels in cells treated with different induction methods showed the same tendency compared to gene expression (Figure 3E and Supplementary Material S3), suggesting improved browning efficiency. These observations indicate that ceiling culture combined with rosiglitazone induced transdifferentiation of beige adipocytes from white adipocytes, resulting in upregulated expression of browning-related genes.

### Ceiling-cultured beige adipocytes display increased expression of fatty acid metabolism genes

**FIGURE 4:**
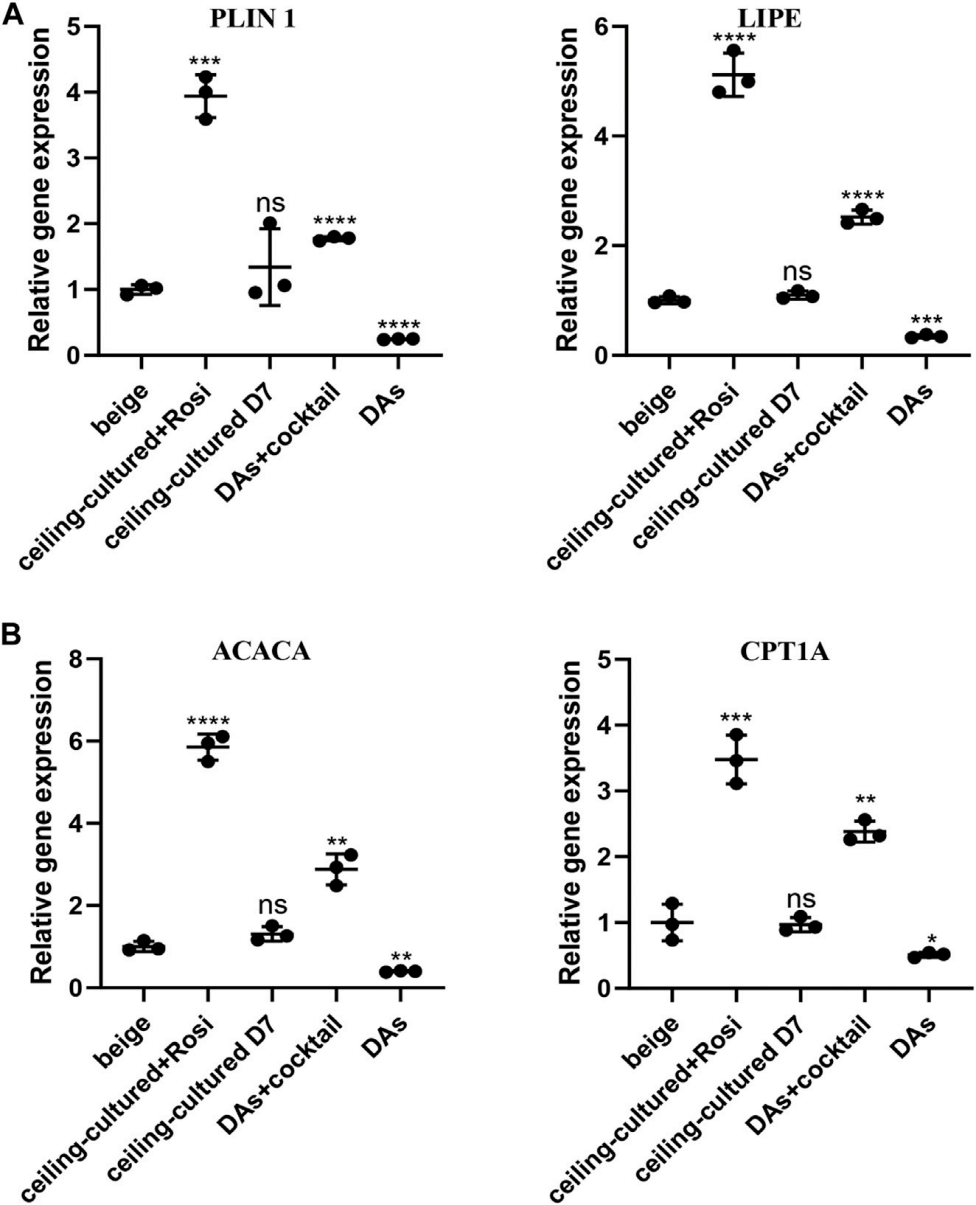
Fatty acid metabolism-related gene expression in beige adipocytes. **(A)** Relative gene expression levels of the lipolysis-related genes *PLIN1* and *LIPE*, normalized to *GAPDH* as an endogenous control. **(B)** Relative gene expression levels of the oxidative genes *ACACA* and *CPT1A*, normalized to *GAPDH* as endogenous control. Data are mean ± SEM (*n* = 3). ns, non-significant. *p < 0.05, **p < 0.01, ***p < 0.001.

We further investigated the role of ceiling culture and rosiglitazone on lipolysis and fat oxidation in mature white adipocytes by measuring the expression of genes involved in lipid metabolism, such as *LIPE* and *PLIN1,* and mitochondrial fat oxidation, including *ACACA* and *CPT1A* (Lone and Yun, 2016). Cells from the ceiling-cultured+rosiglitazone group displayed more LDs, suggestive of lipolysis and supported by elevated expression of lipolysis-related genes *LIPE* and *PLIN1* (Figure 4A). Moreover, expression of *ACACA* and *CPT1A* in ceiling-culture induced beige adipocytes was significantly upregulated upon rosiglitazone treatment, indicating an elevation of fat oxidation (Figure 4B). In addition, DAs+cocktail had higher expression of *LIPE, PLIN1, ACACA and CPT1A,* than classical ADSCs+cocktail beige adipocytes. These results suggest that the increased presence of LDs in ceiling-cultured+rosiglitazone-induced beige cells may be due to increased lipolysis and fatty acid oxidation.

### Beige adipocytes from different sources can be identified by gene expression of cell type-specific markers

**FIGURE 5:**
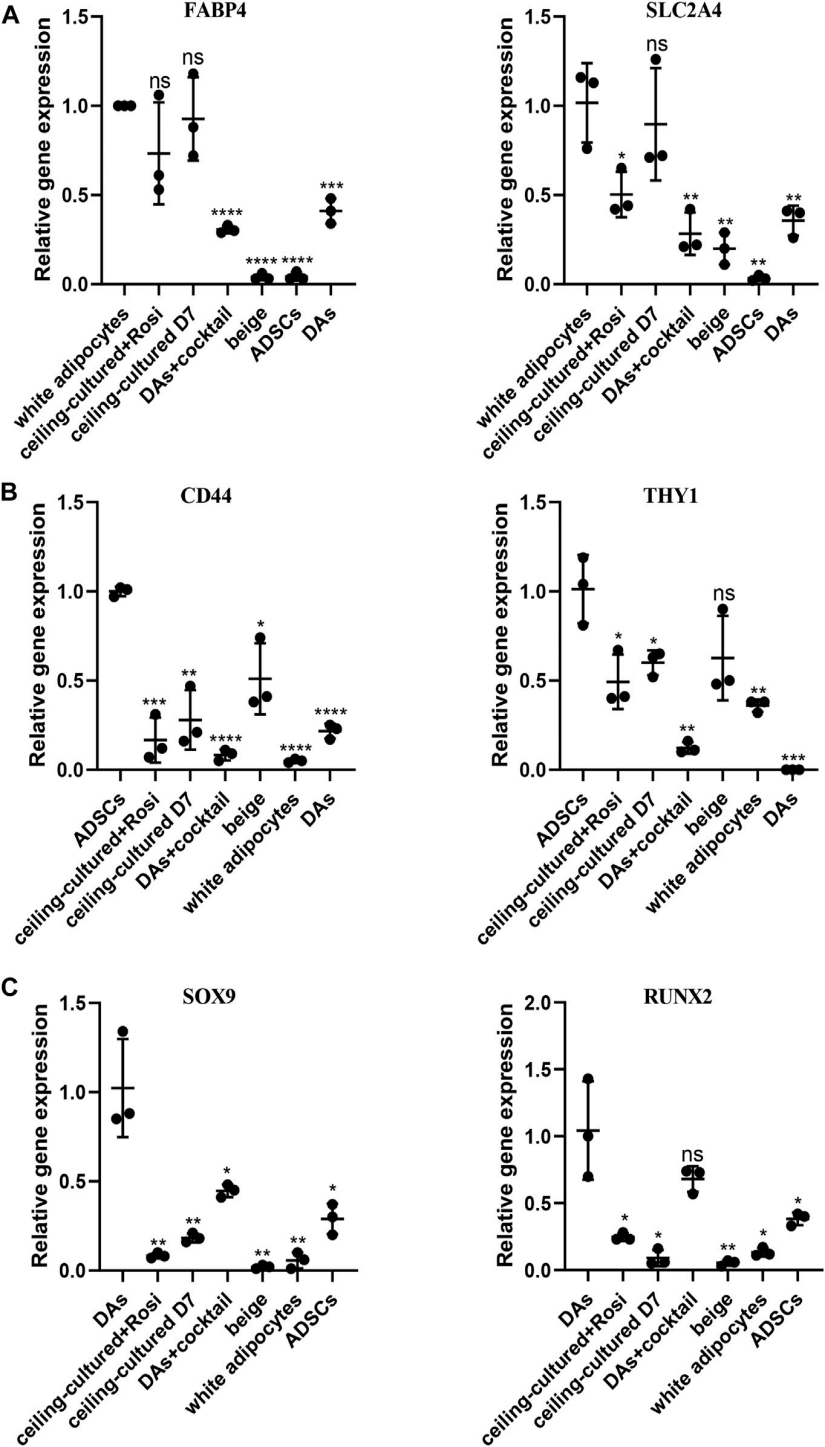
Beige adipocytes from different sources identified by gene expression of cell type-specific markers. **(A)** Relative gene expression levels of adipocyte-specific genes *FABP4* and *SLC2A4*, normalized to *GAPDH* as an endogenous control. **(B)** Relative gene express of the ADSC-specific markers *CD44* and *THY1*, normalized to *GAPDH* as an endogenous control. **(C)** Dedifferentiated adipocytes **(**DAs) gene expression of the mesenchymal lineage-committed markers *RUNX2* and *SOX9*, normalized to *GAPDH* as an endogenous control. Data are mean ± SEM (*n* = 3). ns, non-significant. *p < 0.05, **p < 0.01, ***p < 0.001.

To distinguish between beige adipocytes from different progenitor cells, we quantified several cell markers of gene expression after browning stimulation. During differentiation of human cells, the expression of a progenitor cell’s markers are significantly reduced over time (Lam et al., 2018; Xu et al., 2020). Thus, we selected adipocyte-specific genes as mature white adipose tissue markers, including *FABP4* and *SLC2A4*. There was no significant difference in the expression between ceiling-cultured+rosiglitazone-induced beige adipocytes and untreated ceiling-cultured D7 cells, which are both derived from white adipocytes (Figure 5A). By contrast, high levels of mesenchymal-specific cell key markers CD44, CD90 are expressed on the surface of ADSCs (Najafipour et al., 2019). In line with this, browning cocktail-induced beige adipocytes still expressed higher levels of *CD44* and *THY1* (CD90) than non ADSC-derived cells (Figure 5B). Next, we observed DA-related expression of the mesenchymal lineage-committed marker genes *RUNX2* and *SOX9*. DAs induced by the browning cocktail maintained greater levels of *RUNX2* and *SOX9* expression than beige adipocytes from different progenitor cells (Figure 5C). Together, these results indicate that beige adipocytes cultured from various sources can be identified by gene expression of cell type-specific markers.

## Discussion

Accumulation of excess white adipose tissue contributes to a wide range of diseases, such as obesity, insulin resistance and type 2 diabetes mellitus (Cotillard et al., 2014; Klöting et al., 2010). However, current methods of adipocyte culture *in vitro* are limited in their physiological relevance, which restrict the development of cell and drug therapies for obesity-related diseases. To investigate the pathogenic effects of excess adipose tissue (Skurk et al., 2007), researchers require more functional, responsive cell models. Specifically, a model where mature white adipocytes transdifferentiate into beige adipocytes would more closely simulate processes *in vivo* than differentiation of beige adipocytes from ADSCs.

**FIGURE 6:**
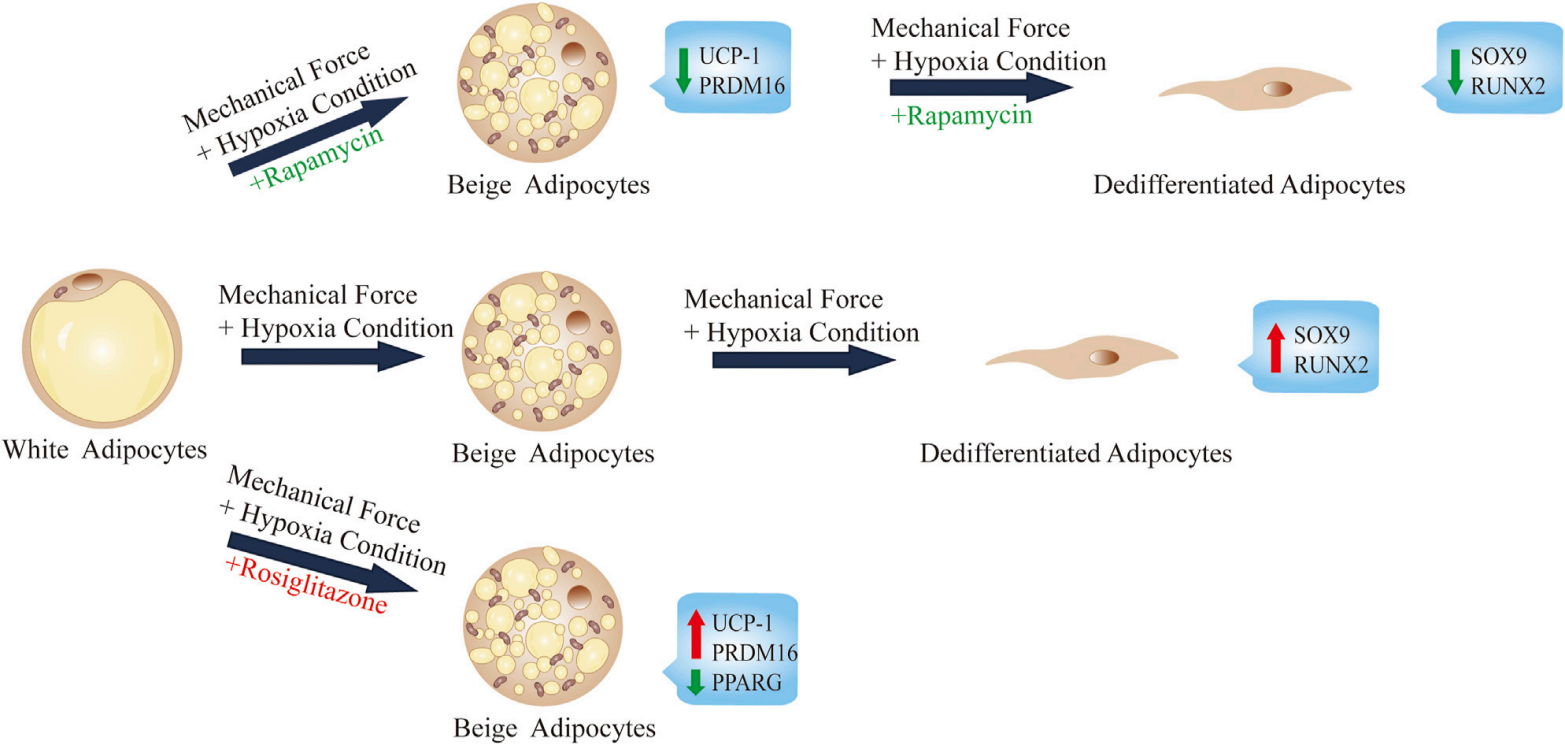
Summary and proposed model of ceiling culture of human mature white adipocytes with a browning agent.

In this study, we show that during the process of adipocyte dedifferentiation in ceiling culture, the continuous mechanical force and browning agent (rosiglitazone) causes adipocytes to accumulate multilocular LDs, similar to those seen in beige adipocytes (Figure 6). In addition, various genes involved in the transcriptional regulation of browning were induced in ceiling-cultured cells: the browning-related genes *UCP1* and *PRDM16* were upregulated, an effect that was suppressed by rapamycin treatment (an inhibitor of browning) in a dose-dependent manner. By contrast, the lipogenic gene *PPARG* was downregulated, and fewer and smaller LDs were present in cells 28 days after seeding in ceiling culture; again, these changes were downregulated in rapamycin-treated cells. Overall, our results suggest that browning may play an important role in the process of dedifferentiation by ceiling culture and that rapamycin appears to prevent this transformation.

Unlike beige adipocytes induced by the classical cocktail, ceiling-cultured beige cells originate directly from mature white adipocytes, which is consistent with the process seen *in vivo*. One challenge in researching browning mechanisms is the lack of models derived from mature white adipocytes, since almost all induction models rely on isolating precursors or stem cells from adipose tissue. After 7 days in ceiling culture, we found that, compared with cells from other sources, gene expression in ceiling-cultured cells was closer to that of mature adipocytes. By contrast, markers of the DAs and ADSCs, the other progenitor cells from which beige adipocytes were derived, maintained their unique, depot-specific gene expression patterns, which further indicates the importance of cell source in creating a physiological model.

The model of transdifferentiating ceiling-cultured white adipocytes into beige adipocytes can be quickly set up, with resulting morphology and gene and protein expression similar to beige cells derived from ADSCs using the classical cocktail induction media. The model has a number of potential applications. Firstly, since ceiling culture more accurately replicates the process of transdifferentiation, this model can be used to study the potential pathophysiological or therapeutic effects of this process *in vitro*. Secondly, we found that the efficiency of browning in ceiling culture was increased with rosiglitazone, but impaired by rapamycin, allowing the relationship between browning and dedifferentiation to be investigated. Thirdly, the environment better replicates the hypoxia and compression present in adipose tissue *in vivo*, especially after fat grafting, providing a suitable model or this procedure. Finally, ceiling culture is a viable model to investigate drug treatment: the presence of beige adipocytes derived from mature white adipocytes may contribute to the resolution of insulin resistance and obesity-associated metabolic diseases. However, exposure of mature white adipocytes under the ceiling culture with hypoxic condition will undergo other cellular responses. Research have reported that the white adipocytes, whether derived from murine or human, exhibit changes over 1300 genes expression in response to hypoxia, including some key adipokines such as leptin, vascular endothelial growth factor (VEGF) and adiponectin (Trayhurn, 2014). In addition, hypoxia also affects the uptake of glucose and increases the production of lactate, with the regulation of genes linked to oxidative metabolism and glycolysis (Trayhurn, 2013). These unavoidable pathways will affect the ultimate productivity of induced beige adipocytes and interfere target for the treatment while stimulating the expression of genes to some extent.

There were some limitations of the study. The role of rapamycin is still controversial. Due to the limitation of chemoresistance of mTOR inhibitor rapamycin, it is still unclear whether the restrain of dedifferentiation *via* inhibition of the mTOR pathway or increase the cytotoxic effects by reactive oxygen species (ROS), apoptosis and mitophagy reportedly(Shen et al., 2018). Therefore, it is less rigorous to restrain the browning by this kind of drugs. As a result, a more specific and effective substitution for rapamycin is required to explore the relationship between browning and dedifferentiation further. Secondly, like other methods, this ceiling culture technology has advantages and disadvantages. The operation is simple, and the culture cycle is short, which makes it a highly alternative culture method. However, there is still a long way to go to accurately control the mechanical pressure intensity and produce functional beige adipocytes in large quantities and high quality in the process of ceiling culture.

Overall, we show that transdifferentiation of white adipocytes to beige adipocytes in ceiling culture is a physiologically relevant model, which may allow the study of phenotypic changes of different adipocytes and provide a novel model for drug screening and the modulation of adipose tissue function.

## Data Availability

The original contributions presented in the study are included in the article/Supplementary Files, further inquiries can be directed to the corresponding authors.
