# Supplementary figures and images for: Ceiling culture of human mature white adipocytes with a browning agent: A novel approach to induce transdifferentiation into beige adipocytes
Source: Front Bioeng Biotechnol. 2022 Aug 15;10:905194. doi: 10.3389/fbioe.2022.905194 (PMC9420896; doi:10.3389/fbioe.2022.905194)

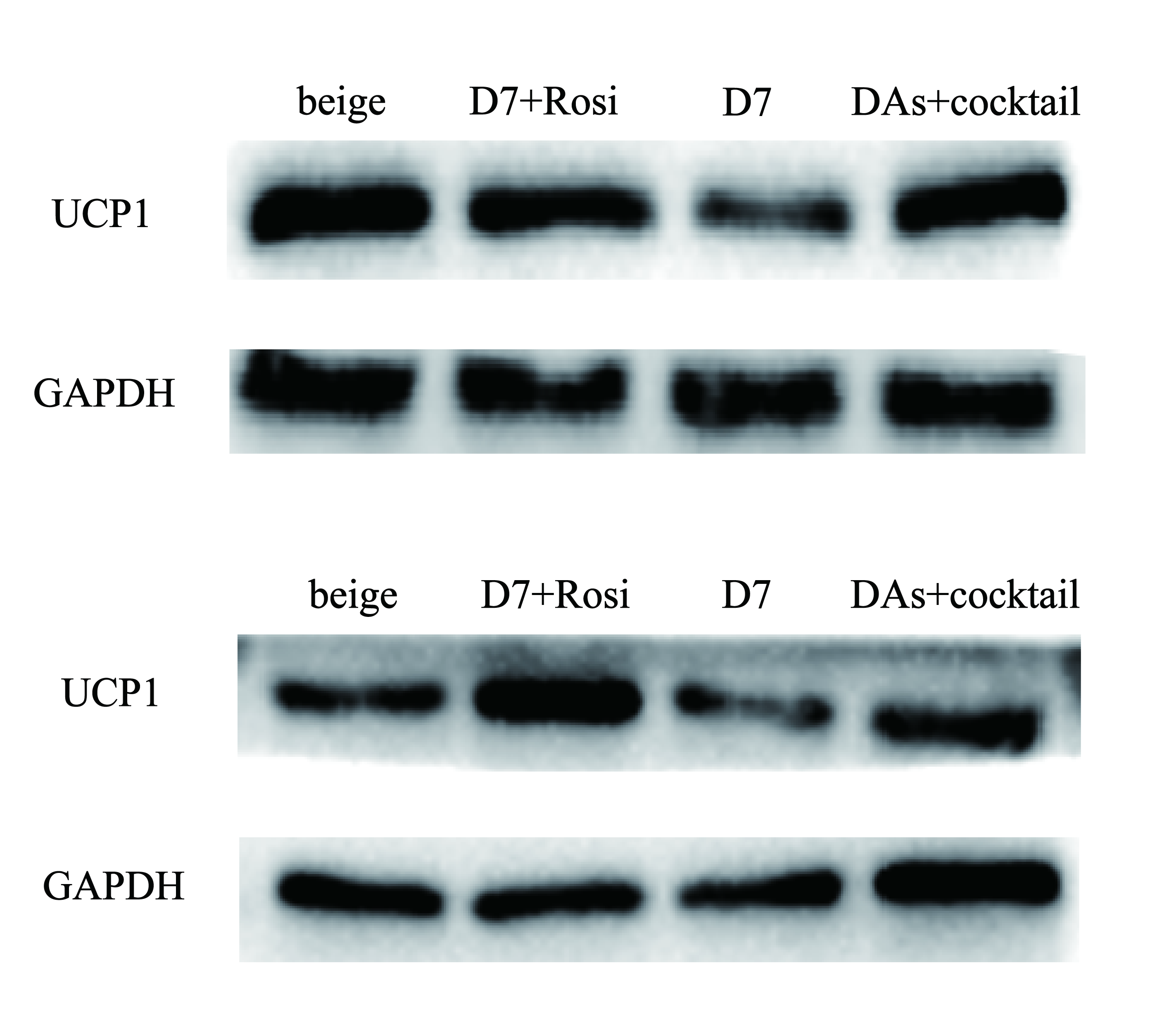

Supplement: Supplementary file 1 [file Image2.TIF]

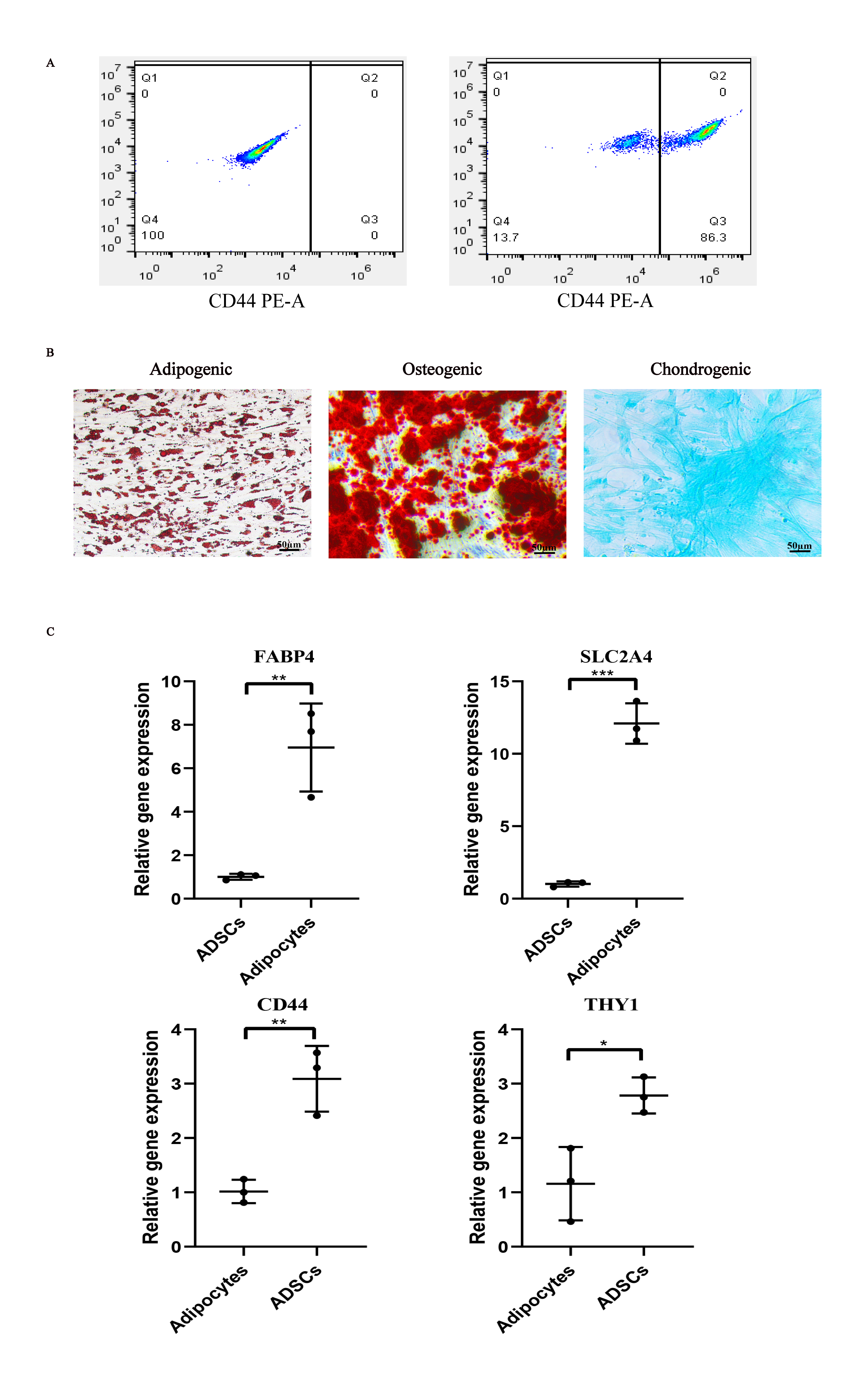

Supplement: Supplementary file 2 [file Image1.TIF]
